# Aspirin and Colorectal Cancer Prevention and Treatment: Is It for Everyone?

**DOI:** 10.1007/s11888-016-0306-9

**Published:** 2016-02-11

**Authors:** Christopher Coyle, Fay Helen Cafferty, Ruth Elizabeth Langley

**Affiliations:** MRC Clinical Trials Unit at UCL, Aviation House, 125 Kingsway, London, WC2B 6NH UK

**Keywords:** Aspirin, Acetylsalicylic acid, NSAID, Colorectal cancer, Chemoprevention, Secondary prevention, Primary prevention, Adjuvant therapy, Bleeding, PIK3CA, BRAF, Single-nucleotide polymorphism, Human leukocyte antigen

## Abstract

There is now a considerable body of data supporting the hypothesis that aspirin could be effective in the prevention and treatment of colorectal cancer, and a number of phase III randomised controlled trials designed to evaluate the role of aspirin in the treatment of colorectal cancer are ongoing. Although generally well tolerated, aspirin can have adverse effects, including dyspepsia and, infrequently, bleeding. To ensure a favourable balance of benefits and risks from aspirin, a more personalised assessment of the advantages and disadvantages is required. Emerging data suggest that tumour PIK3CA mutation status, expression of cyclo-oxygenase-2 and human leukocyte antigen class I, along with certain germline polymorphisms, might all help to identify individuals who stand to gain most. We review both the underpinning evidence and current data, on clinical, molecular and genetic biomarkers for aspirin use in the prevention and treatment of colorectal cancer, and discuss the opportunities for further biomarker research provided by ongoing trials.

## Introduction

Evidence for the anti-cancer effects of aspirin has emerged from in vitro and animal models, epidemiological studies and randomised data, with the most extensive evidence pertaining to colorectal cancer (CRC). Research suggests that aspirin is effective in primary prevention, reducing the risk of adenomas [1] and CRC [2•, 3]. There is also evidence for a possible role in the treatment of cancer, particularly in the adjuvant setting (preventing recurrence and decreasing the likelihood of metastases after potentially curative therapy) [4, 5•].

The potential benefits of aspirin have to be weighed against the risk of adverse effects, particularly in the primary prevention setting. Upper gastrointestinal symptoms (UGS) are a common concern associated with aspirin use, and can limit adherence, but are usually avoidable. The most undesirable effect of aspirin is an increased bleeding tendency, which can manifest as occult gastrointestinal bleeding, epistaxis or purpura. Serious extra-cranial bleeding is rare (an estimated 3.6 additional events per 10,000 people treated for a year with aspirin [6]), with the vast majority of bleeding episodes resolved without sequelae [7•], and intracranial haemorrhage is rarer still (an estimated 0.8 additional events per 10,000 people treated for a year with aspirin [6]).

Identifying biomarkers or clinical characteristics which predict benefit from aspirin use could lead to a more targeted intervention and protect some individuals from unnecessary treatment and possible side effects. A number of potential clinical, molecular and genetic biomarkers have been evaluated including the following: genes mutated in CRC (PIK3CA and BRAF), molecules proposed to have a role in the mechanism through which aspirin exerts its anti-cancer effects (cyclo-oxygenase (COX) enzymes and human leukocyte antigen (HLA) class I expression), and key genetic polymorphisms that may influence the actions of aspirin.

This review will summarise the current data supporting a personalised approach to aspirin use in relation to CRC and highlight the need to discover and validate biomarkers in ongoing trials. The article is structured into four sections: (i) primary prevention of CRC, (ii) treatment of CRC, (iii) safety biomarkers and (iv) other benefit-risk considerations.

## Aspirin and Colorectal Cancer Prevention

The ability of aspirin to prevent colorectal carcinogenesis has been observed in animal models [8–10]. The first clinical evidence emerged in 1988 from a case-control study conducted in Melbourne, Australia, which showed that aspirin reduced the risk of developing CRC [11]. This finding was subsequently corroborated by several other epidemiological studies, with a meta-analysis in 2012 of 30 case-control and cohort studies (*n* = 37,519 CRC cases) showing that aspirin was associated with a lower risk of developing CRC (relative risk (RR) 0.73, 95 % confidence interval (CI), 0.67–0.79) [12]. Two large epidemiological studies have recently provided further supporting data. The Association of American Retired Persons Diet and Health study (AARP) included 301,240 adults aged between 50 and 71 years. An estimated 14 % reduction in CRC was observed with daily aspirin use (hazard ratio (HR) 0.86, CI 0.79–0.94) during 10 years of follow-up [13]. A Danish case-control study of 10,280 CRC cases and 102,800 controls showed a reduction in the risk of CRC (odds ratio (OR) 0.73, CI 0.54–0.99) for those continuously taking aspirin for at least 5 years [14•].

Randomised data substantiate these observations, with a meta-analysis of individual participant data on cancer incidence in randomised trials designed to investigate the effect of aspirin on vascular disease showing that aspirin reduced the 20-year risk of CRC by 24 % (HR 0.76, CI 0.63–0.94), improving to 32 % if taken for ≥5 years (HR 0.68, CI 0.54–0.87) [3]. Similarly, long-term follow-up from the Women’s Health Study (WHS), a randomised placebo-controlled trial designed to assess the effects of aspirin (100 mg on alternate days) in the primary prevention of cardiovascular disease, showed that allocation to aspirin reduced the incidence of CRC by 20 % (HR 0.80, CI 0.67–0.97) [2•].

Recommending aspirin for all has been approached with caution due to the difficulties of predicting the chance of benefit, and the risk of toxicity on an individual level and therefore attention, has naturally turned towards individuals at highest risk of CRC who may benefit most.

## Aspirin and Colorectal Cancer Prevention: Who Benefits?

Groups at the highest risk of developing CRC include those with a hereditary CRC syndrome, a history of colorectal adenomas or an inflammatory bowel disease. Aspirin can exacerbate inflammatory bowel disease and is therefore avoided, but there is evidence to support an effect of aspirin in these other high-risk groups.

The most robust evidence exists for Lynch syndrome (hereditary non-polyposis colorectal cancer), the most common inherited CRC syndrome. The CaPP2 (Cancer Prevention Project 2) trial randomly allocated patients with Lynch syndrome to 600 mg daily aspirin or placebo and found a reduction in CRC incidence in those that remained on aspirin for more than 2 years (HR 0.41, CI 0.19–0.86, *p* = 0.02) [15]. This is in the context of an overall incidence of CRC in the CaPP2 trial population of 5.6 % (48/861) over 4.5 years of follow-up, and is likely to represent the group with the highest absolute reduction in risk of CRC. Intriguingly, a sub-analysis of this trial showed that the increase in risk of CRC associated with obesity can be abrogated by aspirin in this population [16•]. Obesity is, thus, a potential predictive biomarker for aspirin benefit, and further research in populations other than Lynch syndrome is warranted. Less evidence is available to support an effect of aspirin in familial adenomatous polyposis (FAP), another inherited CRC syndrome. The CaPP1 trial randomised patients with FAP (prior to preventive surgery), in a 2 × 2 factorial design, to 600 mg daily aspirin, resistant starch or placebo. They found a trend towards reduced polyp load in aspirin users; however, this did not reach statistical significance (relative risk 0.77, CI 0.54–1.10) [17]. The median duration of aspirin use was only 17 months and it is plausible that a treatment effect may have emerged with longer exposure.

Adenomas are precursor lesions for most cases of CRC, and their prevention or regression has been proposed to represent a surrogate marker for CRC risk [18]. A meta-analysis of four randomised controlled trials, including 2967 individuals with previous adenomas or CRC (without an inherited CRC syndrome), found that those allocated to aspirin had a reduced risk of any subsequent adenoma (risk ratio 0.83, CI 0.72–0.96) or advanced adenoma (risk ratio 0.72, CI 0.57–0.90) [1]. This corresponded to a reduction in the absolute risk of any adenoma of 6.7 % (CI 3.2–10.2 %). Further research is needed to confirm whether individuals with a history of adenomas benefit more from aspirin than those without.

Whilst those at highest risk of CRC are most likely to gain from chemoprevention with aspirin, it may be possible to identify those that benefit in lower risk populations. Newly emerging data show that aspirin users with certain single-nucleotide polymorphisms (SNPs) have a reduced risk of developing CRC, and their absence may describe a group who will not benefit. A case-control study of 840 CRC patients and 1686 matched controls examined CRC risk according to expression of the T allele of rs6983267, which is associated with reduced WNT/β-catenin signalling, a major oncogenic pathway in CRC, proposed to be affected by aspirin. They observed that aspirin reduced CRC risk in the cohort as a whole (OR 0.71, CI 0.60–0.85), but the effect was most marked in individuals with a T allele of rs6983267 (OR 0.83, CI 0.74–0.94) [19••]. A larger case-control study (8634 cases, 8553 controls) examined the risk of CRC according to expression of two different SNPs, rs2965667 (located close to the microsomal glutathione S-transferase 1 gene, often upregulated in CRC) and rs16973225 (located close to the interleukin 16 gene, which has been implicated in CRC carcinogenesis) [20••]. It was observed that use of aspirin and/or non-steroidal anti-inflammatory drugs (NSAIDs) was associated with a reduced risk of CRC amongst individuals with TT genotype of SNP rs2965667 (OR 0.66, CI 0.61–0.70) and the AA genotype of rs16973225 (OR 0.66, CI 0.62–0.71), but not in those with other rarer genotypes. Germline genetic polymorphisms have the potential to identify individuals that benefit from aspirin, as well as those that do not, within populations at lower risk of CRC, and further investigation using existing datasets is required.

## Aspirin and Colorectal Cancer Treatment

**Fig. 1:**
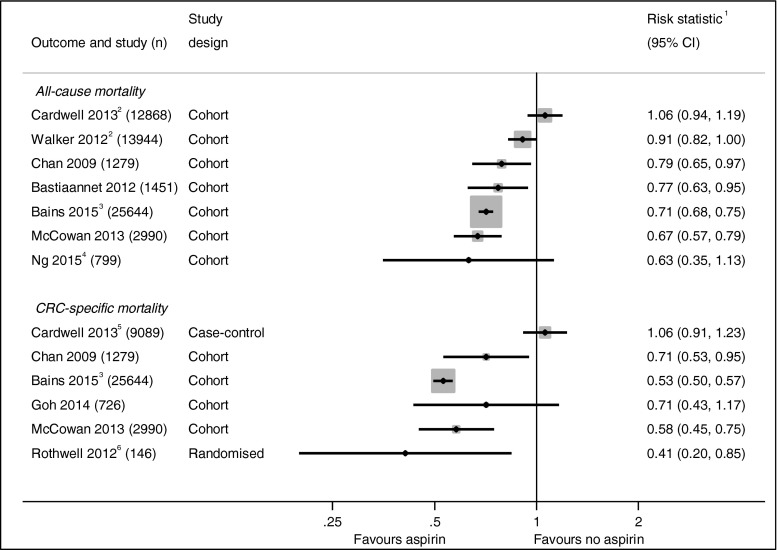
Studies investigating CRC outcomes according to aspirin use following diagnosis. No summary statistic is presented given the high heterogeneity of studies. Multivariate (adjusted) statistics are presented in all cases. *1* All risk statistics are hazard ratios except the studies by Cardwell [23•] and Rothwell [30], which are odds ratios, and Bastiaannet [26] which is a rate ratio. *2* Studies have an overlapping population, both using UK General Practice Data. *3* Published in abstract form only at time of authorship. *4* Cohort taken from randomised chemotherapy trial and is limited to colon cancer. *5* Cohort only matched for CRC-specific mortality analysis. *6* Meta-analysis of cancer outcomes in randomised cardiovascular trials

In vitro studies show that aspirin inhibits proliferation and induces apoptosis in CRC cell lines [21, 22] suggesting a possible role for aspirin in the treatment of CRC. Figure 1 summarises the results of the clinical studies investigating the effect of aspirin use after a CRC diagnosis. The first epidemiological data emerged from the Nurses’ Health Study (NHS) and Health Professionals Follow-up Study (HPFS) showing that regular aspirin use after a diagnosis of CRC is associated with a reduction in CRC deaths (HR 0.71, CI 0.53–0.95) and overall mortality (HR 0.79, CI 0.65–0.97) [24]. This has recently been corroborated by a large cohort of CRC patients from the Cancer Registry of Norway where improvements in overall survival (HR 0.71, CI 0.68–0.75) and CRC-specific survival (HR 0.53, CI 0.50–0.57) were seen with aspirin use after CRC diagnosis [25]. Similar improvements in mortality were observed in data from the Eindhoven Cancer Registry (ECR) (overall survival RR 0.77, CI 0.63–0.95) [26] and in population data collected in Tayside, Scotland (overall mortality HR 0.67, CI 0.57–0.79, and CRC-specific mortality HR 0.58, CI 0.45–0.75) [27]. Observational data from the CALGB 89803 trial (which compared two different adjuvant chemotherapy regimens in patients with stage III colon cancer) has also shown a trend towards improved overall survival (HR 0.63, CI 0.35–1.12) and disease-free survival (HR 0.68, CI 0.42–1.11) in those patients using aspirin both during and after chemotherapy [28•]. A study of 13,994 CRC patients from the UK General Practice Research Database found a strong trend towards a reduction in overall mortality; however, this failed to reach significance (HR 0.91, CI 0.82–1.00) [29].

Data on cancer outcomes from randomised trials investigating the effects of aspirin in vascular disease corroborate the trends seen in epidemiological studies. A meta-analysis which included 13,833 individuals who developed CRC in four vascular trials has shown significant reductions in CRC deaths (HR 0.66, CI 0.51–0.85) [3]. Another meta-analysis of five vascular trials has shown that aspirin is associated with a reduction in the risk of having metastases when CRC is diagnosed (OR 0.36, CI 0.18–0.74) and of subsequently developing them during follow-up when not present at diagnosis (HR 0.26 CI 0.11–0.57) [30].

## Aspirin and Colorectal Cancer Treatment: Who Benefits?

Much of the work on potential biomarkers relating to aspirin use as a treatment for CRC has come from three large cohorts (NHS [24], HPFS [24] and ECR [26]). An initial analysis of the NHS/HPFS dataset reported that the beneficial effects of aspirin on CRC outcomes were restricted to individuals whose tumours overexpressed COX-2 (HR 0.39, CI 0.20–0.76)and not observed for those with weak or absent expression (HR 1.22, CI 0.36–4.18) [24]. Whilst inhibition of either COX-1 or COX-2 is sufficient to inhibit tumourigenesis in mouse models [31], uncertainty exists about the role of COX enzymes in relation to the anti-cancer effects of aspirin, particularly given that the daily doses of aspirin used in vascular prevention are not considered sufficient for sustained COX inhibition in systemic tissues [32]. The predictive utility of COX-2 has not been seen in other colorectal studies [33••] or comparative studies in breast cancer [34].

Although the mechanism by which aspirin exerts its anti-cancer effects remains unknown, one proposed hypothesis was that aspirin, through its anti-platelet effects, could expose circulating tumour cells to immune-mediated destruction by natural killer cells [35] and that this effect would be restricted to tumours with low or absent HLA class I expression. However, analyses of a random sample of colon tumour samples (*n* = 999) from the ECR found that the benefit from aspirin therapy was largely restricted to tumours expressing HLA class I antigens (risk ratio 0.53, CI 0.38–0.74), and was not seen in those who had lost expression (risk ratio 1.03; CI 0.66–1.61) [33••]. This interesting observation, contrary to the original study hypothesis, requires validation in further datasets. HLA class I expression is seen in about a third of colorectal tumours and so could identify a sizeable group who might benefit from aspirin after a CRC diagnosis.

There has been significant interest in the phosphatidylinositol 3-kinase (PI3KCA) gene as a potential biomarker of aspirin response (Table 1). This follows the publication of data from the NHS/HPFS demonstrating that individuals with PIK3CA mutations taking regular aspirin after a diagnosis of CRC had markedly improved CRC-specific survival (HR 0.18, CI 0.06–0.61) compared to those with wild-type tumours (HR 0.96, CI 0.69–1.32). The same association was observed for overall survival (mutated PIK3CA HR 0.54, CI 0.31–0.94, wild-type PIK3CA HR 0.94, CI 0.75–1.17) [36]. This finding was supported by a small ad hoc analysis of the randomised VICTOR trial, where rofecoxib (a Cox-2 inhibitor) was being evaluated after CRC resection but the trial was closed early when rofecoxib was withdrawn from the market. Individuals with PIK3CA mutations taking regular aspirin after diagnosis had improved recurrence rates (HR 0.11, CI 0.001–0.83), whereas those lacking PIK3CA mutation did not (HR 0.92, CI 0.60–1.42), although the number of participants taking aspirin with the mutation was small (*n* = 14) [37•]. However, data from two recent studies has not confirmed the association. In the ECR dataset, the survival benefit associated with aspirin use after a colon cancer diagnosis was seen in those with wild-type PIK3CA tumours (rate ratio 0.55, CI 0.40–0.75), as well as those with PIK3CA-mutated tumours where there was a trend towards a survival benefit but this did not reach statistical significance (rate ratio 0.73, CI 0.33–1.63) [33••]. In addition, in a cohort of patients with PIK3CA-mutated CRCs from the Moffitt Cancer Centre and Royal Melbourne Hospital (*n* = 1487), no overall survival benefit was observed (HR 0.96, CI 0.58–1.57) and, whilst there was a trend towards a CRC-specific survival benefit, this was not significant (HR 0.60, CI 0.34–1.16) [38•]. PIK3CA mutations only occur in 10–15 % of patients with CRC, whereas the epidemiological data suggest that a greater proportion of CRC patients benefit from aspirin use after a CRC diagnosis; therefore, this biomarker needs further investigation, ideally in randomised trials.

**Table 1:** Studies examining PIK3CA mutation, aspirin use and colorectal cancer outcomes

Study	PIK3CA mutation (%)	PIK3CA mutant					PIK3CA wild type				
		No aspirin	Aspirin	Outcome	HR	95 % CI *p* value	No aspirin	Aspirin	Outcome	HR	95 % CI *p* value
NHS and HPFS [36]	16.7	95	66	OS	0.54	0.31–0.94 *p* = 0.01	466	337	OS	0.94	0.75–1.17 *p* = 0.96
				CSS	0.18	0.06–0.61 *p* < 0.001			CSS	0.96	0.69–1.32 *p* = 0.76
VICTOR trial [37•]	11.6	90	14	OS	0.29	0.04–2.33 *p* = 0.19	681	111	OS	0.95	0.56–1.61 *p* = 0.26
				CSS	0.11	0.001–0.83 *p* = 0.027			CSS	0.94	0.59–1.49 *p* = 0.79
MCS and RMH [38•]	12.4	136	49	OS	0.96	0.58–1.57 *p* = 0.86	Study of PIK3CA-mutated tumours only				
				CSS	0.60	0.34–1.16 *p* = 0.14					
ECR^a^ [33••]	15.8	73	27	OS	0.73^b^	0.33–1.63 *p* = 0.4	348	147	OS	0.55	0.40–0.75 *p* < 0.001

Multivariate (adjusted) statistics are presented in all cases

*OS* overall survival, *CSS* colorectal cancer-specific survival, *RFS* recurrence-free survival, *NHS* Nurses’ Health Study, *HPFS* Health Professionals Follow-up Study, *MCS* Moffitt Cancer Centre, *RMH* Royal Melbourne Hospital, *ECR* Eindhoven Cancer Registry, *HR* hazard ratio

^a^Colon cancer only

^b^Rate ratio

A cohort of 1226 patients with a diagnosis of CRC from the NHS and HPFS have also been analysed for BRAF mutation status. The effect of aspirin, after a cancer diagnosis, on cancer-specific and overall survival did not differ according to BRAF mutation status, but may have lacked statistical power. Interestingly, in terms of cancer prevention, aspirin was associated with a lower risk of developing a BRAF wild-type CRC (HR 0.73, CI 0.64–0.83), but not with BRAF-mutated CRC (HR 1.03, CI 0.76–1.38) [39•].

The risk of CRC recurrence after potentially curative treatment depends on a number of prognostic factors, which include stage, mode of presentation, microsatellite instability status and whether adjuvant chemotherapy was administered. Any relative improvement in CRC outcomes with aspirin will need to be considered in the context of an individual’s absolute risk of recurrence.

## Identifying Those at Risk of Toxicity

Aspirin has been used for over 100 years [40] and has a well-documented toxicity profile. Standard contraindications to aspirin use include the following: a history of active or recurrent peptic ulceration, active gastrointestinal bleeding, previous intracranial haemorrhage, a haemorrhagic diathesis or a coagulation disorder. Avoiding co-administration of other NSAIDs, anti-coagulants or corticosteroids also reduces the risk of adverse effects [41].

UGS are common side effects associated with aspirin, with one survey reporting 15.4 % (*n* = 152/986) of long-term low-dose aspirin users experiencing UGS [42]. A history of gastro-oesophageal reflux disease or dyspepsia prior to starting aspirin has been shown to be strongly predictive of UGS on aspirin (OR 17.6, CI 11.52–26.88) [42]. *Helicobacter pylori* infection has been proposed to be a marker of increased risk of developing dyspepsia and a bleeding gastrointestinal ulcer with aspirin [7•]; however, most data supporting this association relates to non-aspirin NSAID use; and therefore, further data is needed to confirm a relationship with aspirin [43]. The HEAT trial (ISRCTN10134725), examining *H. pylori* eradication to prevent ulcer-related bleeding and dyspepsia in aspirin users, is ongoing.

The most common cause for concern in relation to aspirin use is the risk of bleeding. Data from six cardiovascular primary prevention RCTs (*n* = 95,000) estimated that aspirin increased the risk of serious bleeding (excluding intracranial haemorrhage) by 0.04 % per year (from 6.6 events per year in 10,000 individuals to 10.2 events [6]). Age has been shown to be a key predictor of bleeding risk with a recent systematic review estimating that the risk of major bleeding increases between three- and fourfold between the ages of 50–54 and 70–74 years [7•]. Intracranial haemorrhage is even rarer with aspirin estimated to increase the risk by less than 0.01 % per year (from 2.7 events in 10,000 individuals treated for a year in the control groups to 3.5 events in the aspirin groups, HR 1.39, CI 1.08–1.78) in the aforementioned analysis of six cardiovascular RCTs of aspirin. This study also revealed that mean blood pressure is associated with an increased risk of intracranial haemorrhage (rate ratio 2.18, CI 1.65–2.87) [6].

Certain genetic polymorphisms have been proposed as potential biomarkers for NSAID-induced gastrointestinal ulceration and bleeding. A study in a Japanese population (*n* = 480) found that a functional SNP of the COX-1 gene (rs1330344) has been shown to be significantly associated with gastric ulceration (OR 5.80, CI 1.59–21.1) [44]. Additionally, two polymorphisms of CYP2C9 (an enzyme responsible for the metabolism of aspirin) have been found to be significantly associated with bleeding risk in NSAID users [45, 46].

Biomarkers including increasing age, previous dyspepsia and certain SNPs have the potential to identify those at greatest risk of aspirin toxicity. Furthermore, diagnosing and treating conditions like hypertension and *H. pylori* infection could also reduce the change of adverse effects.

## Other Benefit-Risk Considerations

Aspirin is an established treatment for the secondary prevention of cardiovascular disease but is not generally recommended for its primary prevention, however a recommendation for its use in both the prevention of cardiovascular disease and cancer is currently under consideration by the United States Preventive Services Task Force [47]. Little is known about the cardiovascular benefits of aspirin in those with cancer as this is an exclusion criterion in most large cardiovascular trials. CRC and cardiovascular disease have a number of common risk factors, including obesity, high cholesterol and diabetes, and any cardiovascular benefits might add to the rationale for aspirin use in the context of CRC prevention or treatment. Data from cardiovascular trials has additionally suggested that some individuals are resistant to the biological effects of aspirin [48] which also has the potential to limit anti-cancer activity. The existence of aspirin resistance is challenged by the finding that serum thromboxane B2 (a serum marker of platelet activation) is suppressed by aspirin in 99 % of healthy subjects [49]. Other explanations to account for the phenomena include variability in some functional assays of platelet function or undetected poor adherence to aspirin [50]. Fast recovery of platelet function in some individuals might have previously been categorised as resistance, and this might be addressed with twice daily doses [51]. Both the cardiovascular effects of aspirin and the possibility of aspirin resistance could alter the overall risk-benefit profile, and thus require further investigation in existing datasets and ongoing trials.

## Conclusions

The current data on the benefits and risks of prophylactic aspirin in the general population has recently been reviewed by Cuzick et al. who concluded that aspirin use for greater than 5 years (75–325 mg/day), starting between the ages of 55 and 65, has a favourable benefit-harm profile [52••]. Phase III trials investigating the role of aspirin for cancer prevention in the general population are likely to be challenging due to the length of follow-up and number of participants required. However, in higher risk groups, trials are more feasible. The CaPP3 trial (Cancer Prevention Project 3), examining different doses of aspirin for the prevention of Lynch syndrome cancer (ISRCTN16261285), and the seAFOod (Systematic Evaluation of Aspirin and Fish Oil Bowel Polyp Prevention Trial), examining the effect of aspirin and fish oil in patients at high risk of colorectal adenomas (ISRCTN05926847), are ongoing and the results are awaited.

The role of aspirin in the treatment of CRC is also being evaluated in a number of recruiting phase III trials. Add-Aspirin (ISRCTN74358648) is a double-blind placebo-controlled trial for patients with colorectal, breast, gastro-oesophageal and prostate cancer, investigating the effects of aspirin in the adjuvant setting. Participants are randomised to aspirin 300 mg, aspirin 100 mg or placebo for at least 5 years, and it is separately powered for each tumour type to assess the effects of tumour-specific outcomes. Other ongoing trials in this setting include ASCOLT (Aspirin for Dukes C and High Risk Dukes B CRCs, NCT00565708) and ASPIRIN (A Trial of Aspirin on Recurrence and Survival in Elderly Colon Cancer Patients, NCT02301286). There are also three upcoming trials investigating the effects of aspirin in molecularly stratified groups. In the adjuvant setting, ALASCCA is a randomised, double-blind, placebo-controlled phase III trial of aspirin in colorectal patients with mutations in the PI3K signalling pathway (PIK3CA, PIK3R1 or PTEN mutations), and SAKK 41/13 (Swiss Group for Clinical Cancer Research), a double-blind, placebo-controlled randomised trial of adjuvant aspirin treatment in PIK3CA-mutated colon cancer patients (NCT02467582). In the advanced setting, FOCUS4-B (ISRCTN90061546) plans to investigate the role of aspirin in individuals with PIK3CA mutant advanced CRC.

Prior to being considered as an intervention in randomised trials, aspirin has not followed the modern target-driven drug development pathway taken by other anti-cancer agents. Consequently, biomarker discovery and validation is at an early stage. Ideally, predictive biomarkers are co-developed with a drug and validated in phase I and II trials prior to defining the population for phase III trials [53]. However, potential biomarkers for aspirin have mostly emerged from observational studies where there is a risk of confounding and, therefore, it is highly important that they are investigated further in ongoing trials. Research into the mechanisms underlying the anti-cancer effects of aspirin may also reveal new biomarkers. The likelihood of a single biomarker that can identify individuals that will or will not benefit from aspirin is low, and thus, it will be important to investigate patterns of multiple biomarkers to select individuals who will gain from aspirin therapy.

Aspirin is a low-cost, generic agent, available in both resource-poor and resource-rich countries. If it can be shown to be effective, there is the potential for a major impact on the global burden of CRC. Identifying those who are most likely to benefit will be essential to maximising this potential.
